# Automatic measurement of choroidal thickness with swept-source optical coherence tomography in chronic Vogt-Koyanagi-Harada disease: 3 years’ follow-up

**DOI:** 10.1186/s12348-024-00445-7

**Published:** 2024-12-02

**Authors:** Liria Yamamoto-Rodríguez, Alexander Anduaga-Beramendi, Roger Mediavilla-Vallespín, Olaia Subirà, Pere Garcia-Bru, Xavier Solanich-Moreno, Luis Arias, Josep M. Caminal, Olga García-García

**Affiliations:** 1 Ophthalmology Department, University Hospital of Bellvitge, Hospitalet de Llobregat, Carrer Feixa Llarga S/N, Barcelona, 08907 Spain; 2 Internal Medicine Department, University Hospital of Bellvitge, Hospitalet de Llobregat, Barcelona, Spain

**Keywords:** Swept-source-OCT, Vogt-Koyanagi-Harada, Choroid, Thickness, Chronic, Relapses, Indocyanine green angiography

## Abstract

**Purpose:**

The course of Vogt-Koyanagi-Harada (VKH) disease is typically assessed qualitatively using indocyanine green angiography (ICGA) and enhanced-depth imaging optical coherence tomography (EDI-OCT). However, a quantitative approach could improve accuracy and objectivity. The aim of this study was to assess the clinical value of the automated measurement capabilities of swept-source optical coherence tomography (SS-OCT) to measure choroidal thickness (quantitative approach) in chronic VKH.

**Methods:**

Prospective, longitudinal case–control study at a tertiary university hospital involving 23 chronic VKH patients (9 convalescent and 14 quiescent) and 17 age-matched controls. Choroidal thickness was automatically measured with SS-OCT.

**Results:**

Choroidal thinning and improved vision were associated with treatment while increasing thickness and worsening vision were associated with posterior relapse. Overall, 41.6% of asymptomatic (no change in visual acuity) recurrences in the convalescent group and 25% of recurrences in the quiescent group were detected by SS-OCT. All recurrences diagnosed by SS-OCT showed signs of inflammation on ICGA.

**Conclusions:**

Automatic measurement of choroidal thickness with SS-OCT is a rapid, non-invasive method of detecting posterior segment recurrences and treatment response in VKH patients, and could reduce the need for angiography to monitor this disease.

**Supplementary Information:**

The online version contains supplementary material available at 10.1186/s12348-024-00445-7.

## Background

Vogt-Koyanagi-Harada (VKH) syndrome is a noninfectious, bilateral, granulomatous panuveitis that may present alongside neurological symptoms, such as headaches and meningismus, and with auditory symptoms, such as tinnitus and hypoacusia [1–3]. Symptoms differ according to disease duration and stage. VKH syndrome is considered chronic at 12 weeks after onset, where retinal detachment resolves, and cells are cleared from the anterior chamber and the vitreous. Distinctive pigmentary changes in the macula and atrophy of the retinal pigment epithelium (RPE) confer a “sunset glow” fundus. Clinical signs of recurrence following convalescence typically produce anterior granulomatous uveitis and patients may develop dermatologic signs [3, 4].

Indocyanine green angiography (ICGA) is a valuable tool to determine inflammatory choroidal activity in primary stromal choroiditis and to assess choroidal inflammation, regardless of whether or not clinical signs are present. Thus, ICGA is well-suited to detect silent choroidal recurrences^3^. Choroidal inflammation can be seen in ICGA as hypofluorescent dark spots, delayed filling, vessel leakage and hyperfluorescent areas. Nevertheless, ICGA has several disadvantages as an imaging modality. First, it is an invasive, time-consuming technique that requires contrast injection. Secondly, the results are qualitative and thus subject to examiner interpretation. Third, the presence of hypocyanescent spots in choroidal atrophy and inflammation make it challenging to distinguish between stromal choroidal fibrosis and disease recurrence.

Enhanced-depth imaging optical coherence tomography (EDI-OCT) yields in vivo, high-definition, cross-sectional images of the choroid using spectral-domain OCT [5, 6]. Even though several studies have used EDI-OCT to evaluate choroidal thickness in both normal and pathologic eyes [7–11], in up to 26% of eyes, the choroid-scleral boundary cannot be identified with this technique. Consequently, choroidal margins must be delineated manually, which allows for significant inter-observer differences. Therefore, it would be advantageous to have a tool that allows for automatic segmentation of the choroid layer [11–13]. Fortunately, the development of swept-source OCT (SS-OCT) allows for automatic segmentation of the choroid layer. SS-OCT uses a 1-μm band light that penetrates more deeply into the retinochoroidal structures, thus providing better resolution of those layers, particularly in the choroid-scleral interface, making SS-OCT a valuable tool in the assessment of choroidal thickness [14, 15].

Given the time required to perform manual measurements, at our department we use SS-OCT to assess choroidal thickness, which has many advantages over other imaging techniques for determining choroidal thickness. Notwithstanding those advantages, this imaging technique has not, to our knowledge, been previously used to prospectively evaluate patients with VKH. In this context, the aim of the present study was to prospectively assess patients with convalescent/chronic VKH syndrome by means of SS-OCT to measure choroidal thickness over a 3-year period. Changes in choroidal thickness were compared to variations in visual acuity (VA), ICGA findings, and clinical symptoms to determine if there were any significant associations amongst these variables.

## Materials and methods

We prospectively recruited 23 patients with chronic VKH (9 convalescent/recurrent and 14 quiescent) and 17 age-matched healthy controls. The convalescent/recurrent group consisted of patients diagnosed > 12 weeks previously (and therefore still receiving treatment) or patients with a longer follow-up who had experienced a recurrence after treatment completion. Patients whose last treatment was ≥ one year prior to study inclusion and who had no history of recurrences were considered to have quiescent VKH.

Inclusion criteria for patients was an established diagnosis of VKH according to the revised diagnostic criteria for VKH disease [1]. Exclusion criteria (both cases and controls) included any history of ocular disease or surgery, myopia or hypermetropia greater than ± 3 diopters [15], or any history of systemic disease with ocular involvement.

We registered the demographic characteristics of participants before clinical ophthalmic assessment and OCT imaging. A thorough clinical ophthalmic evaluation was made, including best-corrected visual acuity (BCVA) in decimal fractions and Snellen equivalent in feet.

Written informed consent was acquired from all patients in accordance with our institutional guidelines. This study adhered to the tenets of the Declaration of Helsinki. Institutional review board and ethics committee approval was obtained from the Ethics Committee (CEIC) at the Bellvitge University Hospital (reference number PR204/14).

We used ICGA to identify choroidal inflammation in patients with signs of recurrence, or when the SS-OCT showed an increase in choroidal thickness with a simultaneous decline in VA, or when the increase was ≥ 50 µm in the absence of changes in VA. Based on data from other studies on choroidal thickness [8, 9] we obtained ICGA images in all patients with asymptomatic increases in choroidal thickness ≥ 50 µm in order to check for signs of inflammation as this would confirm that the increased choroidal thickness was indicative of a relapse.

### SS-OCT image acquisition protocol

Using a SS-OCT prototype (Atlantis DRI-1 OCT, Topcon, Japan), the macular regions of pathologic and healthy eyes were scanned at a 1,050-nm wavelength and a scanning speed of 100,000 A-scans/second using a high-definition set of 12 radial cuts of 12.0 mm each, horizontal scans, and a cube raster scan protocol (12.0 × 9.0 mm). Retinal, foveal and choroidal thicknesses were automatically acquired. Two choroidal areas were analysed: the subfoveal choroidal thickness (SFCT) and the mean area of the nine measures of the choroidal thickness of the Early Treatment Diabetic Retinopathy Study (ETDRS) grid centred on the fovea (mean grid choroidal thickness: MGCT). The SFCT and the MGCT were used as reference measures. The SS-OCT 3D scan yields a 12 × 9 mm retinal and choroidal thickness map of the macular region following automated delineation of the retina and the choroid. The retinal and choroidal thickness maps were overlapped with the modified ETDRS grid (6 × 6 mm) to obtain computerized measures of SFCT and MGCT. These were then compared with SFCT and MGCT measures obtained with SS-OCT in healthy age-matched controls, both manually and automatically, using the same SS-OCT procedures.

Given that the SFCT was measured in the area of maximum vision, and that previously reported studies have used this measure [12–17], we elected to use the SFCT as the reference value to monitor choroidal thickness during follow up. The number of follow-up consultations in the patients was based on need.

### Definition of relapse

Changes in choroidal thickness were considered posterior recurrences if they were identified simultaneously with decreased VA (after excluding other causes) and/or signs of inflammation on ICGA.

### Statistical analysis

A descriptive analysis was performed to determine mean and median values and dispersion (SD and interquartile range [IQR]). Student’s t tests or the Mann–Whitney U tests were applied to compare the mean values of quantitative variables. All statistical tests were two-tailed. *P* values < 0.05 were considered significant. All analyses were performed with Minitab 17.3.1 software (Minitab, Inc. State College, Pennsylvania, USA).

## Results

Convalescent/recurrent and quiescent patients were followed for up to 3 years (range, 6 to 36 months; mean, 29 months). There were no significant age- or gender-related differences between the groups (Table 1).

**Table 1:** baseline demographic and data of patients with Chronic Vogt-Koyanagi-Harada disease and normal subjects

	**Controls (*n* = 17)**	**Chronic VKH (*n* = 25)**	***p*-value**
Gender, n (%)			
Male	5 (29.4)	10(38.5)	*P* = 0.775
Female	12 (70.6)	16(61.5)	
Age			
Median (Q1Q3)	49 (24–76)	45(38–57)	*P* = 0.328
mean (SD)	49 (15)	46(13)	*P* = 0.2531
Ethnicity			
Caucasian	17 (100)	21 (80.7)	
Hispanic	0 (0)	2 (7.7)	
Asian	0 (0)	0 (0)	
African	0 (0)	3 (11.5)	

### Overall analysis of the study variables

#### Visual acuity (Appendix A)

Posterior recurrent inflammation was found in seven of the nine patients (78%) in the recurrent group, and in three of 14 patients (21%) in the quiescent group. Baseline VA was better than 20/40 in most patients in all groups. Vision improved in 50% of patients in the recurrent group at study end. None of the quiescent patients had worse vision at study end.

#### Choroidal thickness (Appendix A)

There were significant differences in baseline SFCT between the quiescent group and controls (*p* = 0.004), but not between the recurrent and control groups (*p* = 0.327). The quiescent group had a significantly thinner SFCT than controls (*p* = 0.000). No significant differences between choroidal thickness at baseline and study end were observed (*p* = 0.459), indicating that relapses did not affect thickness at the end of follow-up.

Using choroidal thickness in the controls as a reference (220–360 µm), baseline SFCT in the relapse group was normal in six eyes (33.3%), thicker in nine eyes (50%) and thinner in three eyes *(16.6%). At study end, nine eyes (50%) had reverted back to normal choroidal thickness, five (28%) had thinner choroids and four (22%) had a slightly thicker choroid.

There were no significant differences between MGCT and SFCT values amongst the chronic VKH groups (Appendix B), nor between MGCT and SFCT values in the control and VKH groups (Appendix C).

#### Relapses (Tables 2 & 3)

**Table 2:** Analysis of relapses in recurring Vogt-Koyanagi-Harada group: best corrected visual acuity and subfoveal choroidal thickness prior to and at relapse. Number of months at relapse since diagnosis of the illness, and years of follow-up at the end of the study

Patient	VA pre-relapse	VA at relapse	Decrease in vision	SFCT (µm) Pre-Relapse	SFCT (µm) at Relapse	Increase in Thickness	Years since Dx
P1 R1 OD	1	1	NO	323	435	112	7
P1 R2 OS	1	1	NO	340	452	112	
P1 R3 OD	0,9	0,8	YES	92	347	255	
P1 R4 OS	0,8	0,8	NO	90	390	300	
P2 R5 OD	1	0,7	YES	301	454	153	1
P2 R6 OS	1	0,9	YES	346	569	223	
P3 R7 OD	1	0,6	YES	355	570	215	1,5
P3 R8 OS	1	1	NO	394	574	180	
P3 R9 OS	1	0,8	YES	417	438	21	
P3 R10 OD	1	1	NO	359	410	51	
P3 R11 OS	1	1	NO	333	444	111	
P4 R12 OD	1	1	NO	344	394	50	1
P4 R13 OS	1	0,8	YES	313	372	59	
P5 R14 OD	0,8	0,5	YES	350	400	50	19
P5 R15 OD	0,7	0,8	NO	238	384	146	
P5 R16 OS	0,8	0,7	YES	350	389	39	
P5 R17 OD	0,6	0,7	NO	306	383	77	
P5 R18 OS	0,8	0,5	YES	318	380	62	
P5 R19 OD	0,8	0,6	YES	374	393	19	
P5 R20 OS	0,8	0,5	YES	394	453	59	
P6 R21 OD	0,6	0,05	YES	64	90	26	18,5
P6 R22 OS	0,6	0,4	YES	68	73	5	
P7 R23 OD	0,9	0,6	YES	350	567	217	7
P7 R24 OS	0,8	0,8	NO	317	438	121	

*P* indicates patient, *R* Relapse, *OD* right eye, *OS* left eye, *VA* visual acuity (decimal), *SFCT* subfoveal choroidal thickness, *µm* micrometers, *Dx* diagnosis

**Table 3:** Analysis of relapses in quiescent Vogt-Koyanagi-Harada: best corrected visual acuity and subfoveal choroidal thickness prior to and at relapse. Number of months at relapse since diagnosis of the illness, and years of follow-up at the end of the study

Patient	VA pre-relapse	VA at relapse	Decrease in VA	SFCT (µm) Pre-relap	SFCT (µm) at relapse	Increase thickness	Years since Dx
P1 1R OS	1	0.8	yes	210	272	62	18
P4 2R OS	1	1	no	309	357	48	6
P12 3R OD	0,8	0,4	yes	260	285	25	2
P12 4R OS	1	0,9	yes	243	307	64	2

*P* indicates patient, *R* relapse, *OD* right eye, *OS* left eye, *VA* visual acuity (decimal), *SFCT* subfoveal choroidal thickness, *µm* micrometers, *Dx* diagnosis

Of the 10 patients in the recurrent group, one was subsequently excluded due to the diagnosis of systemic erythematous lupus during follow-up. Seven of the 9 patients (78%) in the recurrent group had ICGA-confirmed relapses during the follow up period. The mean increase of thickness was 100 µm in symptomatic eyes and 126 µm in asymptomatic eyes.

The two patients with most relapses (5 and 7, respectively) were the only ones whose baseline and final SFCT was thicker than the largest SFCT in controls (360 µm); the mean SFCT at baseline and at study end in those two patients was 403 µm and 373 µm, respectively. At baseline, three eyes (16.6%) in the recurrent group had a thinner SFCT than controls (< 220 µm); by study end, five eyes (28%) had a thinner SFCT. In the recurrent group, seven eyes (39%) experienced a decrease in thickness from baseline to study end.

Six patients developed a relapse after the VKH diagnosis and prior to the start of this study, as follows: 1 to 1.5 years (*n* = 3), 7 years (*n* = 1) and 19 years (*n* = 2). In the recurrent group, treatment was increased, or the step-down was stopped, when VA decreased or SFCT increased by > 100 µm [18–21].

In the quiescent group, three patients developed a relapse during the study (Table 3). These patients had been diagnosed with VKH 2, 6, and 18 years, respectively, prior to study initiation [22–28]. Only one of these 3 patients was treated for the relapse, which was bilateral with a notable vision loss. In this patient, the relapse occurred one year after completion of treatment versus five and seven years in the other two patients, neither of whom was treated for the relapse, as they showed no decrease in VA. Instead, they were monitored closely and the inflammation resolved within 30 days.

SFCT was within the normal range from the control group (220–360 µm) at the end of the study. No recurrences were observed in patients with less than one year from diagnosis.

There were four cases of anterior uveitis during follow up in the recurrent group (only one with a posterior relapse). In the quiescent group, there was one episode of anterior uveitis in one of the posterior relapses.

#### VA and SFCT at baseline and at study completion (Figs. 1 & 2)

**Fig. 1:**
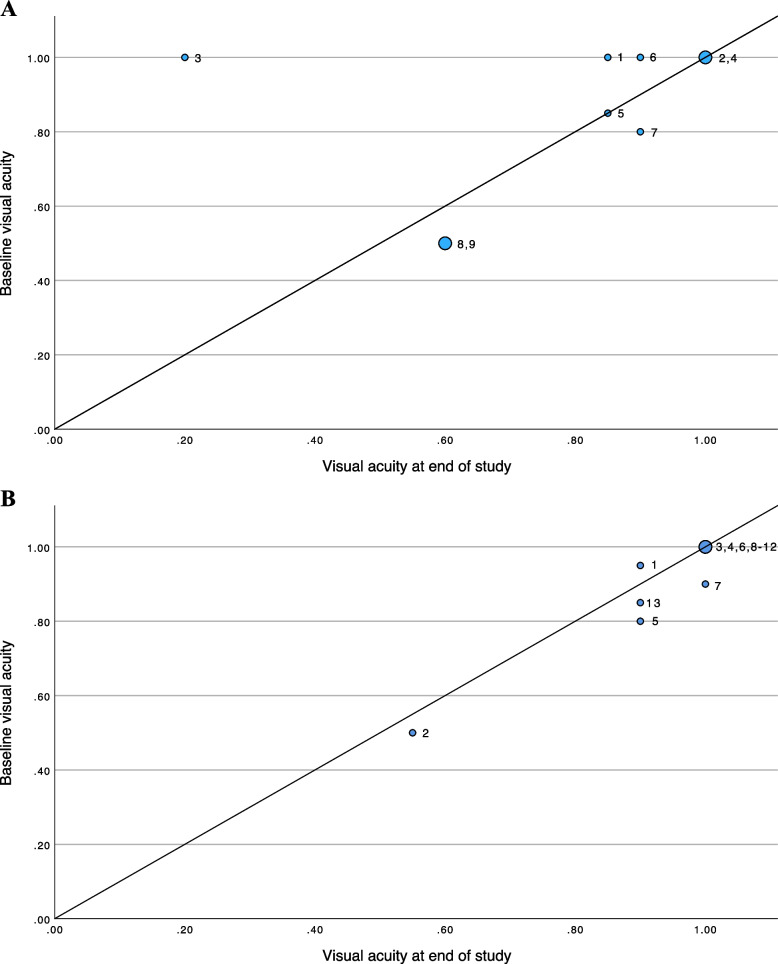
**A** Visual acuity (decimal) at baseline and study end in recurrent group. **B** Visual acuity (decimal) at baseline and study end in quiescent group

**Fig. 2:**
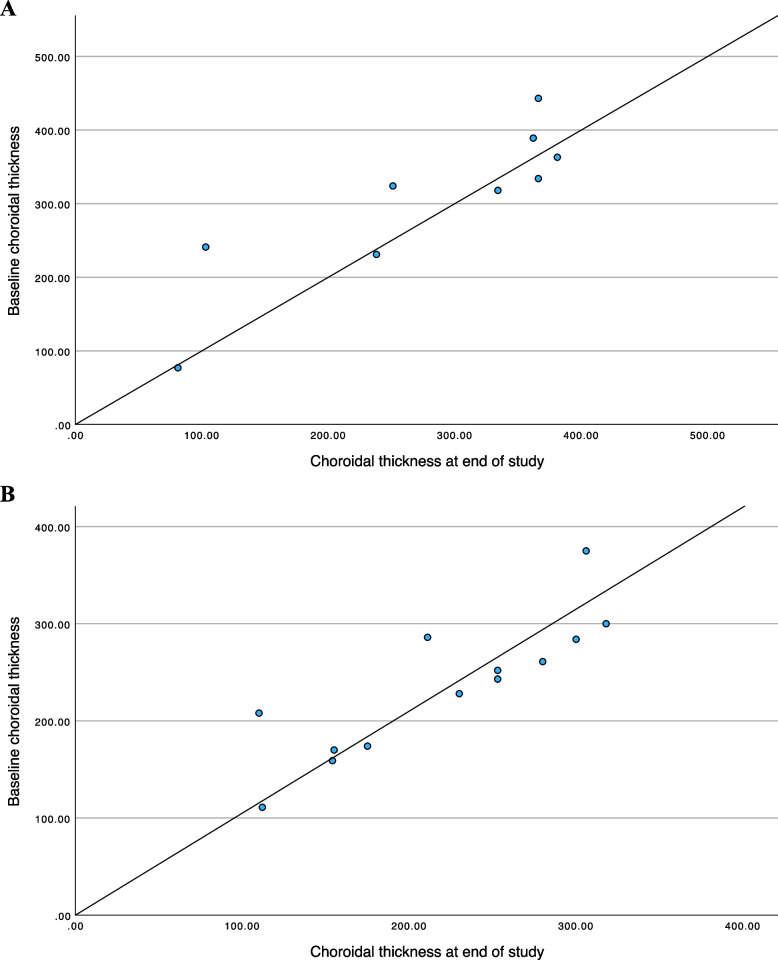
**A** Subfoveal choroidal thickness (micrometers) at baseline and study end in the recurrent group. **B** Subfoveal choroidal thickness (micrometers) at baseline and study end in the quiescent group

Baseline median VA in relapsing VKH was 20/25 (17 eyes); in 10 eyes, vision improved at study completion while vision remained unchanged in the three eyes that had the highest VA. In five eyes (three patients) vision decreased: in one patient (both eyes), the VA was 20/20 at baseline and 20/32–20/25 at the end of follow up; in another patient (both eyes), the VA decreased from 20/25 to 20/50 and from 20/200 to 20/800; in the last patient (one eye), the VA decreased from 20/20 to 20/25.

In the quiescent VKH group, median baseline VA was 20/20 (28 eyes). At study end, none of the eyes with relapses had worse vision than baseline; one eye lost five letters due to an incipient epiretinal membrane.

In the relapse group, baseline SFCT was within the control range in six eyes (33.3%); it was thicker in nine eyes (50%) and thinner in the other three eyes (16.6%). By study end, nine eyes (50%) had recovered normal choroidal thickness, while five (27.7%) had considerable thinning, and four (22.2%) showed slight thickening.

In the quiescent group, SFCT was within normal range (compared to the control group) at baseline and at study end in 64.3% of the eyes. However, SFCT was thinner in 35.7% of the eyes at the start and end of the study. None of the eyes in the quiescent group had thicker choroids at baseline or at study end.

### Examples comparing ICGA information and SS-OCT

**Fig. 3:**
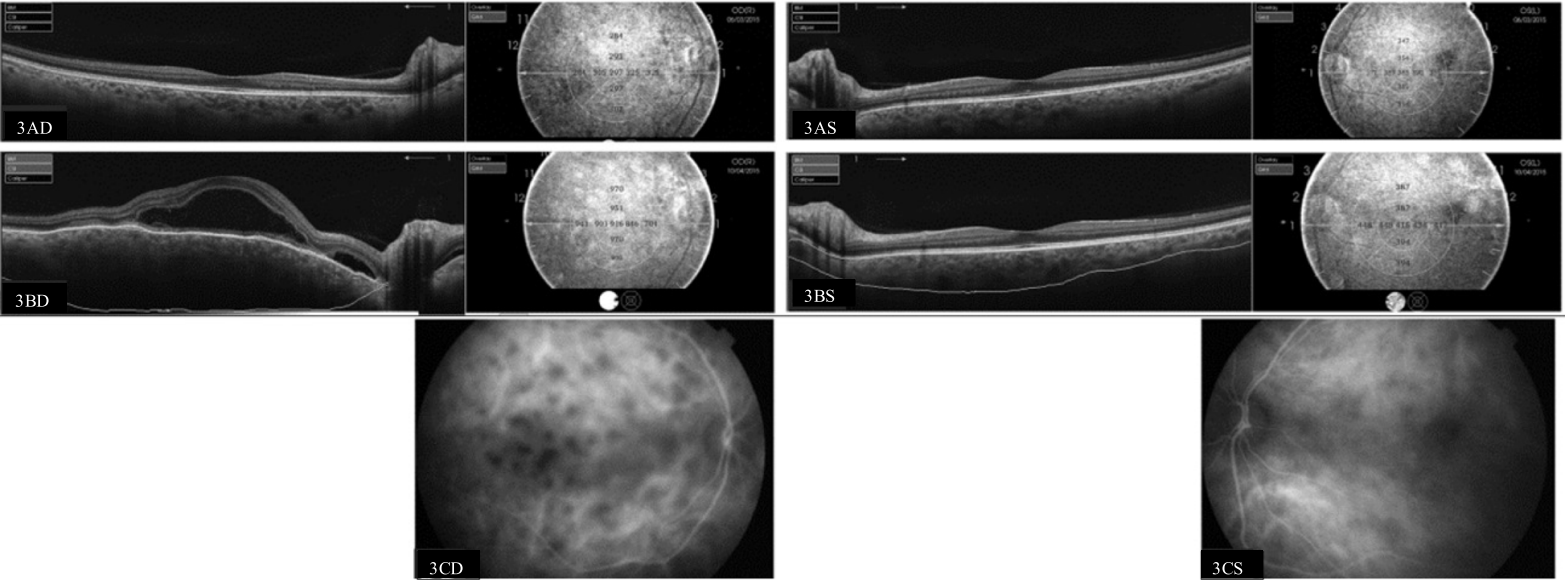
Comparison of swept-source optical coherence tomography (choroid thickness in µm) image and indocyanine green angiography of a symptomatic posterior relapse. 3.A: SS-OCT in March 2015, 3.A.D right eye and 3.A.S left eye: SFCT, 297 µm in right eye and 385 µm in left eye; VA,20/30 in right eye and 20/30 in left eye. 3.B: SS-OCT in April 2015, 3.B.D right eye and 3.B.S left eye: right eye with increased SFCT (916 µm) and multifocal serous retinal detachments and left eye with increased SFCT (416 µm) but no retinal anomalies; VA was 20/200 in right eye and 20/30 in left eye. Bilateral relapse diagnosed with SS-OCT images. 3.C: Indocyanine green angiography of right eye (3.C.D) and left eye (3.C.S) in April 2015: characteristic hypopigmented spots which confirmed choroidal inflammation in both eyes (more in the right eye, with a greater choroidal thickness on SS-OCT)

Figure 3 depicts a bilateral symptomatic relapse with a decrease in VA in the right eye only. The difference in VA can be explained by the amount of inflammatory activity each eye develops. As seen in the SS-OCT images, the choroid in the right eye is thicker than in the left (916 vs. 416 µm), with several multifocal serous retinal detachments and thus a higher degree of inflammation. The ICGA images confirm this finding, showing more hypocyanescent spots in the right eye [29].

**Fig. 4:**
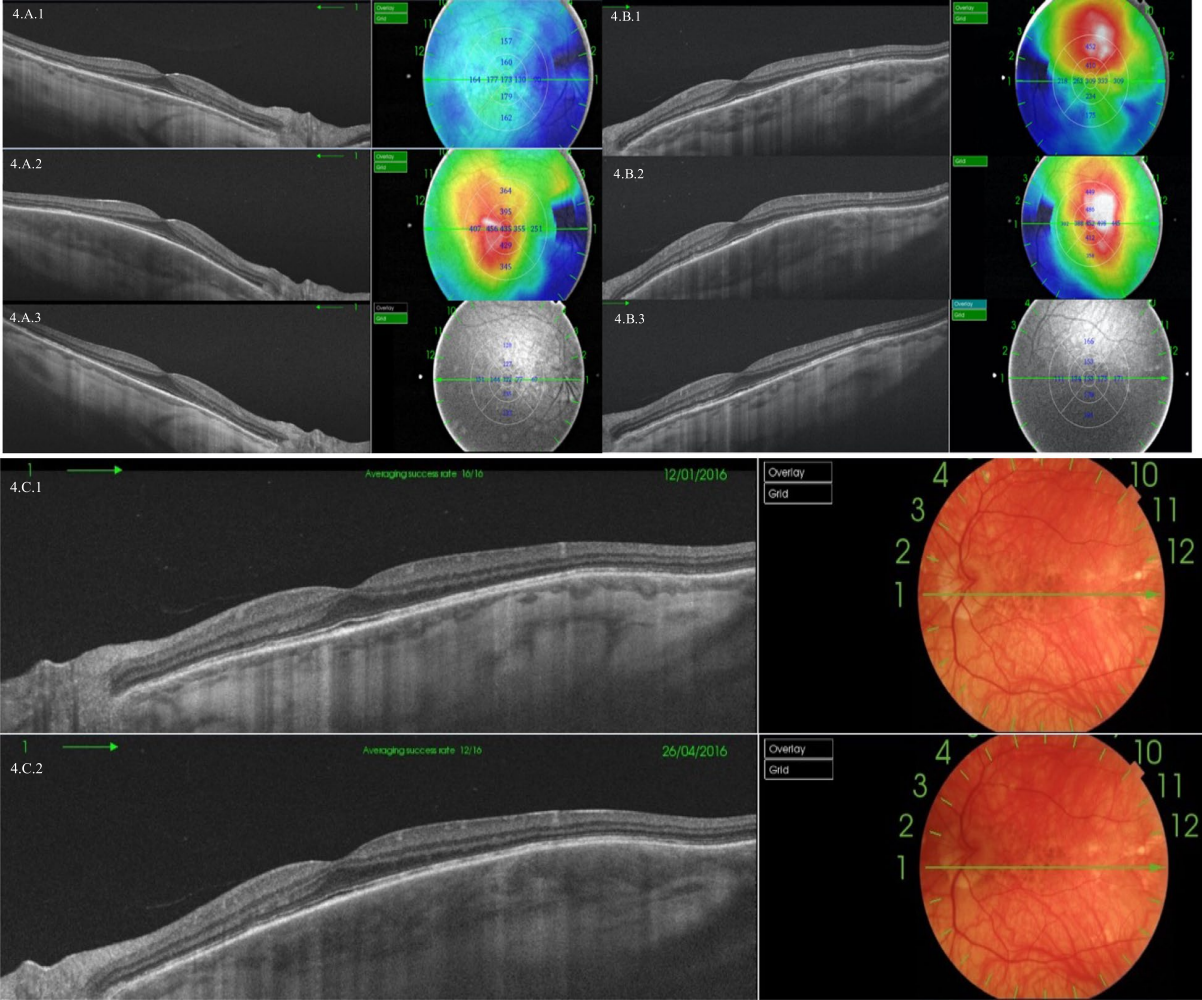
SS-OCT images with choroidal thickness in micrometers (µm) corresponding to VKH quiescent patient with asymptomatic relapses. 4.A.1 SS-OCT of relapse in right eye in January 2014, SFCT 173 µm; 4.A.2: January 2015, SFCT 435 µm; 4.A.3: June 2015, SFCT 122 µm. 4.B.1: SS-OCT of relapse in left eye in January 2014, SFCT 309 µm, Extrafoveal superior Choroidal Thickness 452 µm; 4.B.2: January 2015, SFCT 452 µm, Extrafoveal superior Choroidal Thickness 449 µm; 4.B.3: June 2015, SFCT 153 µm. In 4.B.1 SFCT was normal but extrafoveal superior choroidal thickness was increased. In 4.B.2 SFCT increased to 452 µm. Figure 4.C SS-OCT of right eye. 4.C.1 ss-OCT of the right eye taken in January 2016 and 4.C.2 ss-OCT of the right eye taken in April 2016, showing a change of choroidal thickness in just 3 months. SS-OCT Fundoscopic images show classic chronic VKH signs like peripapillary atrophy, foveolar PRE hyperplasia(^), sunset glow fundus and spot-form scars due to choroidal atrophy in previous Dalen Fuchs granulomas(*)

Figure 4 provides an example of an asymptomatic posterior relapse in a quiescent patient. The figure shows three posterior relapses. The extreme thinning of the choroid in a non-myopic patient makes this finding quite remarkable. In the absence of inflammation, the choroid measures 173 µm in the right eye vs.122 µm in the left eye.

**Fig. 5:**
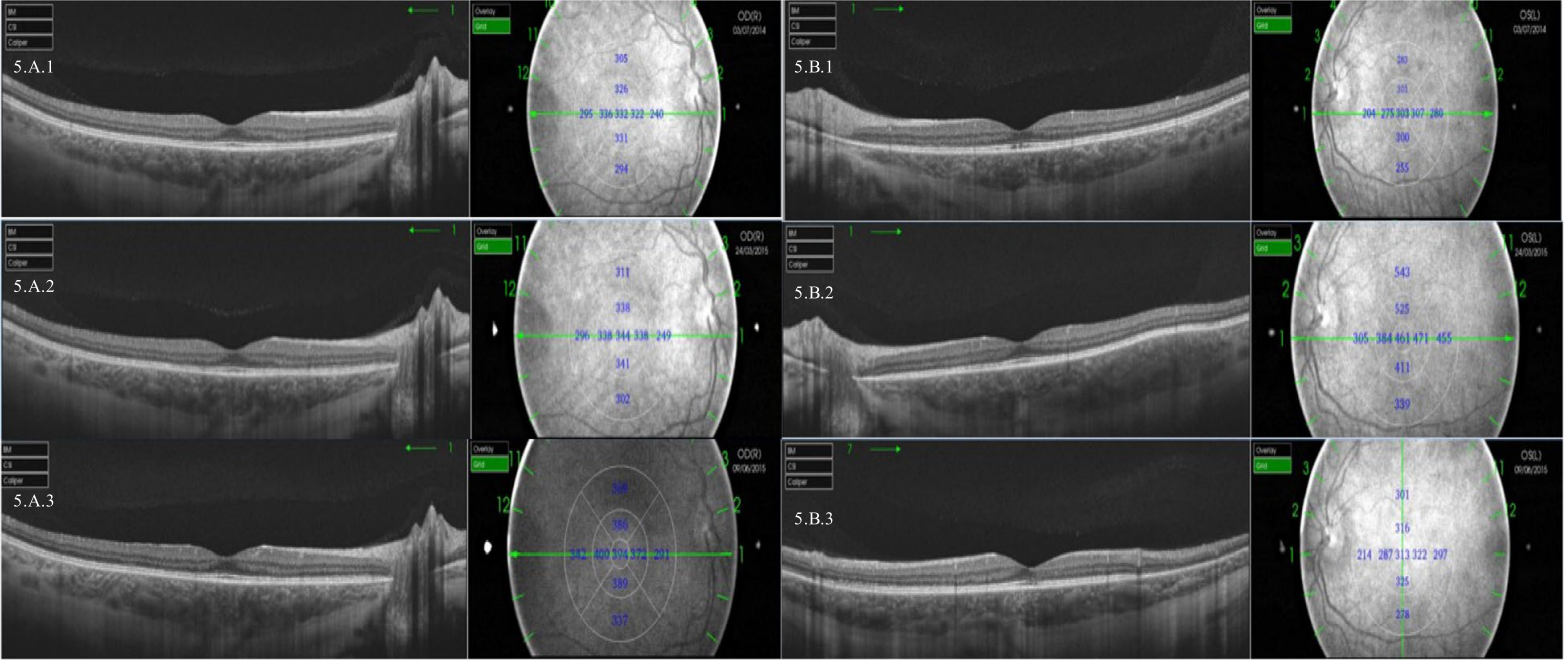
SS-OCT images with choroidal thickness in micrometers corresponding to a patient with recurrent VKH. Both eyes had a total of 3 posterior recurrences, 2 of which were asymptomatic. The images compare choroidal thickness before and in the asymptomatic posterior relapses. 5.A.1: July 2014, SFCT 332 µm, VA 20/20. 5.B.1: July 2014, SFCT 303 µm, VA 20/20. 5.A.2: March 2015, SFC5 344 µm VA 20/20. 5.B.2: March 2015, SFCT 461 µm VA 20/20. 5.A.3: June 2015, SFCT 394 µm VA 20/20. 5.B.3: June 2015, SFCT 313 µm VA 20/20

Figure 5 shows SS-OCT images from a patient with relapsing VKH who had three posterior relapses (two asymptomatic). In these relapses, the loss of VA was not associated with the increase in choroidal thickness.

**Fig. 6:**
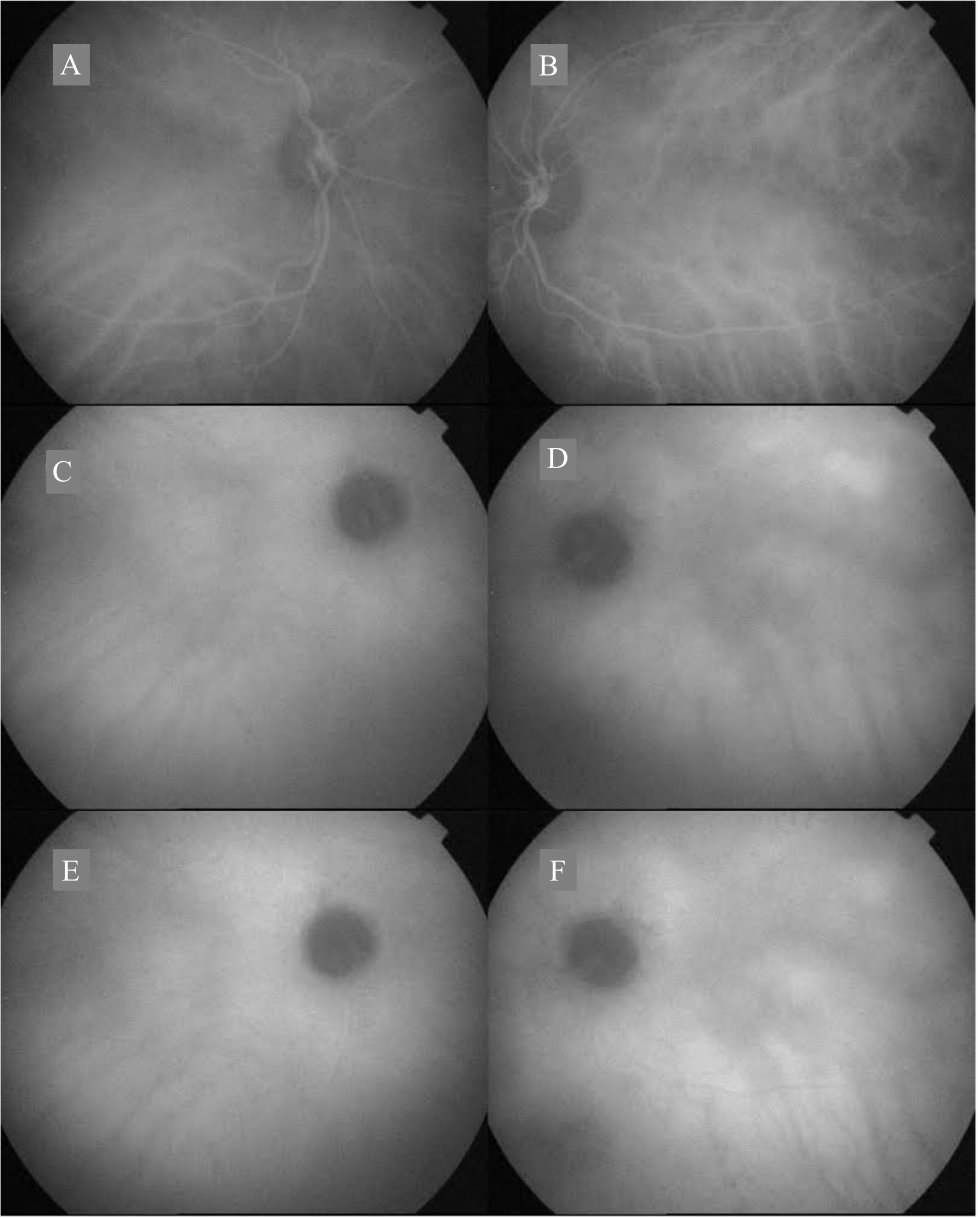
Indocyanine green angiography in an asymptomatic relapse. **A**-**D**, and **E**, **F** we observed at first minutes, 15 min and 30 min respectively. Hypofluorescent spots at the beginning and increased clearance and hyperfluorescence of the same area at the end of the angiography are observed. Inflammation signs match with choroidal thickness in superior temporal retina of Fig. 5 (image 5.B.2)

Figure 6 shows an ICGA image of the asymptomatic posterior relapse of the left eye of the same patient depicted in Fig. 5, with an increased choroidal thickness involving the superior temporal retina seen in Fig. 5B.2. The signs of inflammation revealed by ICGA confirm the relapse diagnosed by SS-OCT imaging.

## Discussion

Given the disadvantages of ICGA and EDI-OCT, alternative approaches to assessing the course of VKH disease are needed. In this study, we assessed the clinical value of the automated measurement capabilities of SS-OCT to measure choroidal thickness (quantitative approach) in the follow up of patients with chronic VKH (convalescent and quiescent patients). We found that choroidal thinning and improved vision were associated with treatment, while increasing thickness and worsening vision were associated with posterior relapse. During follow-up, increases in choroidal thickness and worsening VA were associated with posterior relapse. Overall, 41.6% of asymptomatic (no changes in visual acuity) recurrences in the convalescent group and 25% in the patients with quiescent VKH were detected by SS-OCT. Importantly, in the recurrent VKH group, 14.6% of relapses were also asymptomatic and in the quiescent VKH group 25% of the relapses were asymptomatic. By contrast, all relapsed eyes showed signs of inflammation on ICGA. Moreover, we found the importance of measuring extrafoveal choroidal thickness as well to detect recurrences. Our findings suggest SS-OCT may be a valuable adjunct to ICGA to detect posterior segment recurrence and to evaluate treatment response in patients with VKH disease [20, 21]. All recurrences diagnosed with tomography showed signs of inflammation on ICGA. These results are consistent with a previous study by our group in patients with acute VKH [22] in whom no changes in VA were observed in over half of eyes in which relapses were diagnosed by SS-OCT. Finally, these results are also in line with several other previous studies [19–21].

We and several other groups [22, 23, 26–28] have found that choroidal thickening, which may be related not only to inflammatory infiltration but also to increased exudation^18^, is often but not exclusively found in acute VKH. The results of the present study suggest that chronic relapsing cases also present choroidal thickening. An important advantage of the present study and the aforementioned studies is that those studies did not evaluate choroidal thickness over time as a follow-up measure.

We hypothesized that we could use SS-OCT to measure changes in choroidal thickness to detect posterior relapses. In asymptomatic patients with recurrent VKH, the first sign of relapse was an increase in choroidal thickness (detected by SS-OCT) without loss of VA (all relapses were confirmed with ICGA). Thus, although ICGA is necessary to confirm the relapse, SS-OCT can detect recurrent disease without the need for this invasive, time-consuming procedure. Moreover, while ICGA provides a qualitative diagnosis of choroidal inflammation, SS-OCT yields an objective, quantitative diagnosis of that inflammation (evidenced by the increase in choroidal thickness). Notably, personalized follow-up with SS-OCT scanning allowed us to diagnose pathologic increases in choroidal thickness in all eyes with posterior recurrences (even asymptomatic ones), all of which were subsequently confirmed by ICGA. These results are in line with the findings of our previous study [22] in acute patients, in which SS-OCT was used to detect asymptomatic posterior recurrences, similar to previous reports [22, 23, 27, 28, 30–33].

The tendency for choroidal thickness to decrease in convalescent/chronic VKH patients has been described by several other authors [19, 23, 30, 34]. In our study, the decrease was seen after relapse, but we found no significant differences between choroidal thickness at baseline and at study end in either the relapsed or quiescent groups, indicating that these relapses did not affect choroidal thickness. However, in quiescent patients, choroidal thickness was significantly thinner than in controls at both the baseline and final evaluation. Furthermore, we are unaware of other articles having published any cases of choroidal thickening starting from such thin choroids as the ones illustrated in Fig. 4. It was previously thought that thin choroids could not thicken pathologically even in the event of inflammation, or that even if they thickened, they could not surpass the thickness of a healthy choroid. Nevertheless, this case shows how even an extremely thin choroid can thicken up to 435 µm during a relapse. Interestingly, this case also shows how despite noteworthy choroidal thickening, VA was unaffected. It also exemplifies how posterior relapses do not necessarily have to involve the foveal region. Therefore, it is imperative to determine choroidal extra-foveal thickness using SS-OCT to detect any possible asymptomatic relapses.

Nakayama et al. [26] evaluated eight patients with a recent diagnosis of VKH. In that study, the authors measured choroidal thickness manually with EDI-OCT, defining a recurrence as an increase in choroidal thickness > 100 µm from previous measurements. In our study, we defined the relapse as the minimum increase in thickness required to induce a decrease in VA and the presence of inflammatory signs on ICGA, or without any loss of vision but with an increase ≥ 50 µm in choroidal thickness combined with the presence of inflammatory signs on ICGA. The 50 µm cut off point was based on the mean SD in the various published studies [8, 10, 30, 35]. Nakayama and colleagues found that VKH patients had an increased SFCT at baseline, with a mean increase of 578 μm vs. 666.9 μm in acute patients in our previous study [36]. By contrast, baseline SFCT in the present study was within the normal range in healthy controls (302.3 μm); 302 μm in the relapsing group and 243 μm in the quiescent one. Nakayama et al. also found that choroidal thickness decreased with treatment, consistent with our findings. However, they also found a rebound in choroidal thickening in three patients (5 eyes) during corticosteroid tapering but without evidence of increased inflammation (at one year follow-up with manual measurements). By comparison, in our study, five patients (10 eyes) in the acute group, five patients (10 eyes) in the recurrent group, and one patient (one eye) in the quiescent group presented asymptomatic relapses. We suspect the higher relapse rate observed in our study versus Nakayama et al. may be due to our longer follow-up (36 vs. 12 months). Moreover, our results are largely consistent with previous studies [9, 10, 22, 23, 26, 30, 37, 38].

Nishisho et al. [35] evaluated short-term changes in choroidal structure following adalimumab treatment for refractory, non-infectious uveitis, finding that using EDI-OCT, the SFCT appeared to be an effective choroidal imaging biomarker especially in VKH disease. SFCT decreased significantly from baseline to the values observed at two months (*p* = 0.007).

We did not find any correlation between the amount of visual loss and the increase in choroidal thickness in the patients with recurrent VKH. Generally, our results suggest that patients with more relapses and a thinner choroid will have a worse VA than patients with fewer relapses and a thicker choroid, presumably due to loss of tissue integrity with each additional relapse. Thus, choroidal thickening does not necessarily seem to imply a worsening of VA in a patient with few relapses. In addition, recurrences did not seem to affect the patients’ final VA, regardless of whether the patient was in the relapsing or quiescent group; by contrast, patients in the acute group had a better VA at study end.

We found a higher percentage of posterior recurrences than other published studies, probably because we were able to detect increases in choroidal thickness that were previously overlooked. In addition, our findings show that asymptomatic relapses tend to decrease over time.

When a relapse was detected in the recurrent group, in most cases the step-down treatment was discontinued, however, treatment was increased in some patients depending on the extent of vision loss and the degree of inflammation. Step-down treatment was resumed once choroidal thickness and VA were normalised compared to previous values. In the quiescent group, we restarted treatment in only one patient with decreased VA and choroiditis, which resolved by the next follow up visit. None of the other patients required treatment.

Finally, it is well-known that the presence of a hypopigmented fundus (sunset glow) could be attributable to unidentified (and thus, untreated) posterior recurrences [39], which is why routine use of ICGA during follow up is recommended in VKH patients^3^. However, given the aforementioned drawbacks of ICGA, the use of this modality should be minimized. In this regard, monitoring SFCT with SS-OCT may offer a valuable complement to ICGA. Although we used ICGA to confirm the presence of inflammation in all cases of relapse, we found that SS-OCT was quicker, easier and safer than angiography. Although we do not know how many asymptomatic relapses the patients may have had prior to inclusion in the study, the high rate of sunset glow fundus observed was not unexpected given that asymptomatic relapse appears to be associated with sunset glow fundus.

### Study strengths and limitations

The main limitation of this study is the small sample size. Another limitation is that—despite the recurrent nature of this disease—we did not perform standardized monthly follow-up appointments in all patients. Rather, examinations were individualized according to the severity of each case. Another potential limitation is the cut-off value of ≥ 50 µm before performing the ICGA, as this may have led to an underdiagnosis of recurrences; however, this cut-off was selected to limit ICGA choroidal assessments to avoid performing unnecessary angiographies.

To our knowledge, this is the first study to use the automated measurement features of SS-OCT to measure choroidal thickness in patients with recurrent and quiescent VKH. Moreover, this prospective study includes the largest sample to date of Caucasian, Western European patients with VKH. Although other studies [23, 26, 30] have measured choroidal thickness in acute and chronic VKH, those studies were conducted in Japanese patients using EDI-OCT or SS-OCT [19] with manual measurement. Ours is the first study to present data on automated—and therefore objective—measurement of choroidal thickness obtained during the course of this disease, and also the only one that includes quiescent patients.

## Conclusions

The results of this study suggest that the automatic measurement of choroidal thickness using SS-OCT can be a valuable tool to evaluate treatment response and diagnose posterior recurrences. If these findings are confirmed, SS-OCT could replace ICGA in many clinical scenarios, thus reducing the need for ICGA in routine follow-up. Angiography may only be necessary in asymptomatic patients who develop an increase in choroidal thickness. However, this finding must first be confirmed in large, prospective studies.

## Supplementary Information

Supplementary Material 1.

## Data Availability

No datasets were generated or analysed during the current study.

## Abbreviations

VKH: Vogt-Koyanagi-Harada
ICGA: Indocyanine green angiography
EDI-OCT: Enhanced-depth imaging optical coherence tomography
SS-OCT: Swept-source optical coherence tomography
RPE: Retinal pigment epithelium
VA: Visual acuity
BCVA: Best corrected visual acuity
SFCT: Subfoveal choroidal thickness
ETDRS: Early Treatment Diabetic Retinopathy Study
MGCT: Mean grid choroidal thickness
