# Supplementary material for: Automatic measurement of choroidal thickness with swept-source optical coherence tomography in chronic Vogt-Koyanagi-Harada disease: 3 years’ follow-up
Source: J Ophthalmic Inflamm Infect. 2024 Dec 2;14:62. doi: 10.1186/s12348-024-00445-7 (PMC11612052; doi:10.1186/s12348-024-00445-7)
Supplement: Supplementary file 1 — Supplementary Material 1. [file 12348_2024_445_MOESM1_ESM.docx]

**Appendix A**

| Variables | Healthy controls (n = 34) | **Recurrent VKH** (baseline) (n =18) | p-value* (controls vs VKH baseline) | Recurrent VKH (study end) (n =18) | p-value* (controls vs VKH study end) | p-value* (baseline vs study end) |
| --- | --- | --- | --- | --- | --- | --- |
| **Visual acuity** | | |  |  |  |  |
| Mean (SD) | 1.0 (0.0) | 0.79 (0.27) | p=0.0007 | 0.76 (0.27) | p=0.000 | p=0.5823 |
| Median (Q1-Q3) | 0.9 (0.7 - 1.0) | 0.8 (0.55-1) |  | 0.8 (0.55-0.9) |  |  |
| **SFCT** | | |  |  |  |  |
| Mean (SD) | 302 (71) | 302 (102) | p=0.3271 | 272 (108) | p=0.8415 | p=0.4654 |
| Median(Q1-Q3) | 315 (240 - 350) | 317 (240 - 374) |  | 322(181 - 358) |  |  |

VKH indicates Vogt-Koyanagi-Harada, *Mann-Whitney’s U Test, SD: standard deviation, decimal visual acuity, SFCT subfoveal choroidal thickness, choroidal thickness measured in micrometers, Q1-Q3 Quartile 1 and Quartile 3.

| Variables | Healthy controls (n = 34) | **Quiescent VKH** (baseline) (n =28) | p-value* (controls vs VKH baseline) | Quiescent VKH (study end) (n =28) | p-value* (controls vs VKH study end) | p-value* (baseline vs study end) |
| --- | --- | --- | --- | --- | --- | --- |
| **Visual acuity** | | |  |  |  |  |
| Mean (SD) | 1.0 (0.0) | 0.92 (0.14) | p=0.0121 | 0.94 (0.13) | p=0.0784 | p=0.5419 |
| Median (Q1-Q3) | 0.9 (0.7 - 1.0) | 1 (0.9 - 1) |  | 1 (0.925 - 1) |  |  |
| **SFCT** | | |  |  |  |  |
| Mean (SD) | 302 (71) | 243 (77) | p=0.0045 | 226(75) | p=0.0003 | p=0.4593 |
| Median(Q1-Q3) | 315 (240 - 350) | 245 (169 - 299) |  | 227(158 - 295) |  |  |

VKH indicates Vogt-Koyanagi-Harada, *Mann-Whitney’s U Test, SD: standard deviation, decimal visual acuity, SFCT subfoveal choroidal thickness, choroidal thickness measured in micrometers, Q1-Q3 Quartile 1 and Quartile 3.

**Appendix B**

| Mid grid choroidal thickness (MGCT) versus subfoveal choroidal thickness (SFCT) in chronic VKH patients. | | |
| --- | --- | --- |
| **Variables** | **MGCT No. 25** | **SFCT No. 25** |
| Mean (SD) | 282.7 (130.9) | 305.0 (136.0) |
| Median (Q1-Q3) | 265.0 (193.0 - 340.0) | 291.0 (221.0 - 381.0) |
| Missing | 1 (3.8%) | 1 (3.8%) |

T-test p-value=0.5496

| Mid grid choroidal thickness (MGCT) versus subfoveal choroidal thickness (SFCT) in quiescent VKH patients. | | |  |
| --- | --- | --- | --- |
| **Variables** | **MGCT No. 14** | **SFCT No. 14** | |
| Mean (SD) | 232.1 (69.2) | 252.9 (79.7) | |
| Median (Q1-Q3) | 237.0 (190-266.5) | 277.5 (183.2 - 294.8) | |
| Missing | -- | -- | |

T-test p-value=0.4675

| Mid grid choroidal thickness (MGCT) versus subfoveal choroidal thickness (SFCT) in recurring VKH patients. | | |  |
| --- | --- | --- | --- |
| **Variables** | **MGCT No. 9** | **SFCT No. 9** | |
| Mean (SD) | 347.1 (163.5) | 371.3 (165.8) | |
| Median (Q1-Q3) | 340.0 (250 - 474) | 387 (276.5 - 480.0) | |
| Missing | 1 (8.3%) | 1 (8.3%) | |

T-test p-value=0.7273

**Appendix C**

**Table 1.** **Baseline subfoveal choroidal thickness in control group and chronic VKH patients.**

|  | n | Average | SD | p-value* |
| --- | --- | --- | --- | --- |
| Control* | 17 | 290 | 71 | -- |
| Chronic VKH | 25 | 305 | 136 | 0.6302 |

DE: desviació estàndard; *T-test mostres independents

VKH: Vogt-Koyanagi-Harada, SD: Standard deviation. *We compared the subfoveal choroidal thickness of the control group in our study with the control group of the Ruiz-Medrano et al study (n=276, average 301.89, SD= 80) using a t-test analysis. The results show a difference in average subfoveal choroidal thickness of 11.89 (CI95% -14.27 – 38.05), which is statistically non-significant (p-value = 0.3718).

**Table 2. Baseline mid grid choroidal thickness in control group and chronic VKH patients.**

|  | n | Average | SD | p-value* |
| --- | --- | --- | --- | --- |
| Control | 17 | 276.7 | 69 | -- |
| Chronic VKH | 25 | 282.7 | 130.9 | 0.84 |

VKH: Vogt-Koyanagi-Harada, SD: Standard deviation *T-test for independent samples.

**Table 3. Final subfoveal choroidal thickness in control group vs quiescent and recurring VKH patients.**

|  | n | Average | SD | p-value | p-value |
| --- | --- | --- | --- | --- | --- |
| Control* | 17 | 290 | 71 | -- | -- |
| Quiescent VKH | 14 | 218.93 | 73.13 | 0.0076^2^ | 0.1784^1^ |
| Recurring VKH | 9 | 280.78 | 114.39 | 0.8332^3^ | -- |

SD: Standard deviation;

1) T-test: quiescent vs recurring;

2) T-test: control vs quiescent;

3) T-test: control vs recurring.
